# Education Research: The Complex Case Conference (CCC): A Multispecialty Conference to Enhance Clinical Collaboration Within Neurology

**DOI:** 10.1212/NE9.0000000000200376

**Published:** 2026-11-12

**Authors:** Harli Weber, Galina Gheihman, Gabriel Rodriguez, Tracey Milligan, Stephanie Gandelman

**Affiliations:** 1 New York Medical College, Valhalla, NY;; 2 Department of Neurology, Mass General Brigham, Boston, MA;; 3 Harvard Medical School, Boston, MA;; 4 Department of Surgery, Westchester Medical Center, Valhalla, NY;; 5 Institute of Public Health, New York Medical College, Valhalla, NY;; 6 Department of Neurology, New York Medical College, Valhalla, NY; and; 7 Department of Neurology, Westchester Medical Center, Valhalla, NY.

## Abstract

**Background and Objectives:**

Clinical neurology is becoming increasingly subspecialized, yet neurologists regularly encounter challenging, undifferentiated cases that span subspecialty boundaries. Few formalized opportunities exist for neurologists to present complex cases to peers with differing expertise, especially in multisite departments. Our study had 2 aims: (1) to assess whether there was a perceived need for a formalized mechanism for cross-specialty case discussion in a single multisite institution and (2) if so, whether a monthly, hybrid, structured case conference could meet this need.

**Methods:**

To address our first aim, we completed a needs assessment by surveying neurology residents and attendings at a multisite academic medical center. We then addressed our second aim by implementing the Complex Case Conference (CCC); an 18-month follow-up survey was conducted to evaluate its utility and impact. We used a “Plan, Do, Study, Act” quality improvement approach and report changes across cycles. We report descriptive statistics; comparative analyses used the Fisher exact test and Wilcoxon rank-sum tests.

**Results:**

Twelve respondents completed the needs assessment. In the preceding month, 75% (9/12) had at least 1 case where multispecialty input would have been helpful, yet 33% (4/12) reported having a good mechanism to discuss challenging cases with other subspecialties or within their own subspecialty (33% [4/12]). CCC was developed to address these needs. CCC was held monthly for 60 minutes, during protected Grand Rounds time, with 30 minutes dedicated to a preselected case and 30 minutes as an open forum. Twenty responses were included in the postimplementation survey. The proportion of respondents who felt they had a good mechanism to discuss challenging cases significantly increased, with other subspecialties (33% [4/12] to 80% [16/20] [*p* = 0.036]) and within their own (33% [4/12] to 90% [18/20] [*p* = 0.002]). Respondents also reported increased knowledge of existing institutional resources (17% [2/12] to 70% [14/20]) [*p* = 0.009]). There was no significant change in intradepartmental referrals or recognition of cases where multispecialty input would be beneficial.

**Discussion:**

After an institutional needs assessment, CCC was developed to foster multispecialty clinical collaboration within an academic department. It provides a dedicated forum for discussing cases that would benefit from multispecialty input and has proven valuable for complex patient discussion, supporting clinician growth, and sharing institutional knowledge.

## Introduction

Clinical neurology is becoming increasingly subspecialized, with more trainees pursuing fellowship training after completing their general neurology residency training.^1,2^ It is estimated that over 90% of trainees graduating from neurology residency pursue one of the 31 diverse accredited and nonaccredited subspecialties within neurology.^2-4^ Although this trend toward subspecialization helps develop clinicians with deep expertise in an area of focus, it is also contributing to the growing shortage of general neurologists.^5^ At the same time, given high rates of innovation across the field of neurology, the breadth of knowledge required to care for patients with neurologic conditions is expanding rapidly.^6^ It is difficult for any general neurologist or subspecialist to stay appraised of all the changes happening across the field from which their patients may benefit.

The trend toward subspecialization poses a paradox: although in-depth knowledge in a particular area is essential later in the diagnostic and treatment pathway of neurologic diseases, most patients with neurologic concerns initially present undifferentiated and may benefit from a clinician with broader knowledge and expertise for initial workup and diagnosis. Even those patients who present with seemingly clear clinical pictures often have needs that cross-subspecialty boundaries. Furthermore, this subspecialization places potentially unnecessary burdens on patients who may be required to visit multiple different subspecialists within the same field for their diagnostic workup and care.^7^

The importance of multispecialty, interspecialty, and multidisciplinary collaboration is no secret in neurology. Many academic institutions have weekly or monthly multispecialty case conference series to help bring subspecialties together to discuss complex cases and advance patient care. For example, epilepsy surgery case conferences include faculty in epilepsy, neurosurgery, and neuroradiology,^8^ and stroke case conferences include faculty in vascular neurology, neuro-interventional, and neuroradiology. Although these conferences undoubtedly offer fruitful discussion and often lead to advancement in patient care, many of the cases discussed already have a diagnosis and focus on next steps in therapeutic management.

In addition to multispecialty conferences, some institutions practice interspecialty collaboration, for example, joint efforts between cardiology and neurology through Structural Heart-Brain clinics and boards to evaluate patent foramen ovale (PFO)–associated strokes.^9^ Although these joint neurology and cardiology clinics and conferences have shown to reduce the number of clinic visits for patients before PFO closure decision is made, they do not aid in diagnosis of patients with complex conditions.^9^

In addition, multidisciplinary clinics centered around care for a common condition are well established across the field of neurology, with notable examples in the literature for amyotrophic lateral sclerosis (ALS), multiple sclerosis, and headache.^10-12^ These clinics are another example of how multiple different skill sets come together to advance patient care, providing patients with a one-stop opportunity to see multiple providers including subspecialized neurologists, physical, occupational, speech therapists, and social workers. Centers have shown that these multidisciplinary clinics increase patient satisfaction,^13^ and specifically in ALS, multidisciplinary clinics have been associated with improved prognosis.^14^ In addition, in some subspecialty areas such as headache, these multidisciplinary clinics have been correlated with reduction in headache days and disability.^12^ Although these models have proven to be useful, patients often present to these clinics after the diagnostic mystery has been solved.

Although these established examples of multispecialty, interspecialty, and multidisciplinary collaboration through case conferences and clinics have been shown to reduce patient burden and improve communication within the field of medicine as a whole, there are few reports of departments finding ways to manage patients whose needs cross neurologic subspecialties specifically when a diagnosis is not known.

To address these challenges and their impact in our diverse neurology department, our study had 2 specific aims: (1) to assess whether there was a perceived need for a formalized mechanism for cross-specialty case discussion at a single, multisite institution, via a needs assessment within the Department of Neurology, and (2) If so, whether the existence of a monthly hybrid, structured case conference—the Complex Case Conference (CCC)—could meet this need.

Specifically, the CCC was designed as a multisubspecialty conference, rather than a multidisciplinary or interspecialty conference, where neurologists of all neurologic subspecialties along with trainees are encouraged to come together to discuss challenging patient cases that attending physicians feel they could use the help of colleagues from other subspecialties.

Here, we describe the approach and outcomes of both research aims: first, the needs assessment, and second, the development and implementation of the CCC and the results from a postimplementation survey after 18 months. We approached this work through a quality improvement lens, with iterative Plan, Do, Study, Act (PDSA) cycles. Our experience offers a novel approach to managing complex general neurology cases that may benefit from broader expertise.

## Methods

### Study Design

We approached this work using a quality improvement methodology, including a local needs assessment followed by implementation of the initial version of the CCC and subsequent iterations through PDSA cycles. The needs assessment addressed our first aim, determining whether there was a perceived need for a formalized mechanism for cross-specialty case discussion in a single, multisite institution. This informed the development of the CCC. Our second aim was to assess whether the CCC met this need, evaluated with a postimplementation survey. We used an unmatched preintervention/postintervention analysis. Statistical significance was defined as a *p* value less than 0.05.

### Setting and Participants

The needs assessment, CCC implementation, and evaluation took place within the Department of Neurology at Westchester Medical Center (WMC) and the WMC Health Network (WMCHealth) in Valhalla, NY, USA. WMC is a large, 888-bed regional health system with a high case-mix index that serves as the only academic medical center and main referral center for the Hudson Valley region.^15^ The neurology department is spread over 1 suburban site and 3 suburban-rural sites.

At the start of the study, the department had 24 attendings as well as 9 residents total across the PGY2, PGY3, and PGY4 classes. During the study period, both the size of the residency program and number of neurology faculty grew considerably, resulting in a larger pool of participants in the follow-up survey.

All residents and attending physicians in the Department of Neurology were invited to participate in the surveys and to participate at CCC conferences once implemented.

### Preimplementation Needs Assessment

A needs assessment survey was conducted with residents and attending physicians to evaluate the perceived need and desire for a place for faculty and trainees to discuss challenging cases with experts in the various subspecialties within neurology. The survey instrument was developed de novo for the purposes of designing the case conference curriculum (see eAppendix 1 for full survey). The preimplementation needs assessment survey was administered to all residents and attendings through the online SurveyMonkey platform (SurveyMonkey Inc., San Mateo, CA) from December 2023 to January 2024. The survey was conducted anonymously.

After the need was identified based on the initial survey, the study was submitted to the Investigational Review Board (IRB) and deemed exempt from review and without need for formal informed consent.

### CCC Design and Implementation

A prespecified cutoff for need was not determined. However, given that the needs assessment survey demonstrated that a majority (>50%) of respondents agreed they did not have a good mechanism for multispecialty case discussion and a majority also felt that they could identify a patient case that month that would have benefitted from such discussion, 2 authors (SG and TM) who led the survey determined this was a sufficient demonstration of local need to trial the implementation of the CCC and evaluate whether this format could sufficiently meet the need for multispecialty case discussion. The CCC was implemented in January 2024, and the study was conducted between January 2024 and June 2025, during which 18 total conferences took place.

CCC was designed as a monthly conference, scheduled in place of protected time for departmental Grand Rounds. All department faculty and trainees across all the institutions within the health network are encouraged to attend CCC either in person or virtually. To further encourage faculty participation, conferences take place on the weekly academic half day for the department and faculty were able to receive CME credit for attendance.

The CCC conference is 60 minutes in length and divided into 2 parts (Figure). The first 30 minutes are dedicated to a single faculty presentation of a preselected case; cases that have either a diagnostic or therapeutic question that would benefit from discussion by the group. In preparation, faculty create a presentation to detail the patient's diagnostic journey, including notable history, pertinent positive and negative aspects of the presentation, serial physical examinations, relevant diagnostic tests, and therapeutic trials up to present.

**Figure:**
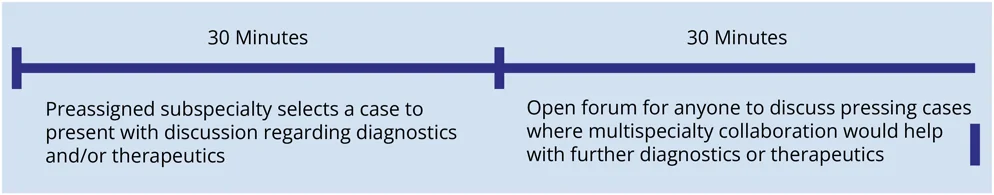
Complex Case Conference (CCC) Session Design A graphical representation of how time is allotted within a single CCC session.

Each division of the department is assigned 1 month of the CCC during the year and 1 faculty member from the division volunteers or is selected to present. This structure ensures that all divisions have equal opportunity to use the forum and that acute in-hospital cases do not overshadow outpatient cases evolving at a slower pace.

During the presentation, a CCC moderator guides the forum as attendings, residents, and students across the department discuss the case. The presenter may pose specific questions for the group, such as what may fall in the differential diagnosis for a certain neuroimaging finding. Conference participants offer suggestions, comments, and ideas to help advance the patient's care. At the end of the 30-minute case presentation and interactive discussion, the moderator summarizes the conversation and details the list of “next steps” for the patient's care.

After the structured, prepared case presentation, the second 30 minutes of the CCC serve as an open forum for anyone to discuss pressing active cases where multiple perspectives may benefit patient care. Cases may range from in-hospital critical care to outpatient cases. During this time, the moderator helps keep the discussion focused and summarizes concrete takeaway points.

### Postimplementation Survey

Eighteen months after the implementation of the CCC, a postimplementation survey was administered to all residents and attendings within the department, regardless of whether they personally presented during the CCC or participated as an audience member. The same questions as posed in the pre-implementation survey to the entire department were repeated to assess for changes in access to discussions within one's own specialty and across specialties in the department. In addition, we asked open-ended questions to understand respondents' perspectives on how input from colleagues helped with determining next steps in diagnosis or management of their patient. We also inquired about factors influencing attendance to the newly implemented conferences and the experience of preparing the case with a mentor/mentee, if applicable. The survey was sent by email and was anonymous and voluntary.

To provide transparency to study participants, the postimplementation survey highlighted that the survey was part of a research project, completely voluntary, and entirely anonymous without any expected benefits to participation in the research. The first question of the postimplementation survey asked participants if they had read and understood the above information and made the informed decision to participate in the survey. If selected “yes” the survey would continue, however if selected “no” the survey ended at that point (see eAppendix 2 for full survey).

### Data Analysis

Demographics were summarized with descriptive statistics. We conducted an unmatched preintervention/postintervention analysis. To evaluate for significant differences, we used the Fisher exact test and Wilcoxon rank-sum test. Statistics were performed using R (version 4.3.1, R Foundation for Statistical Computing, Vienna, Austria). Statistical significance was defined as *p* < 0.05. Using descriptive thematic analysis, 1 author (G.G.) categorized free responses into key themes, which were reviewed and approved by the entire research team.

### Standard Protocol Approvals, Registrations, and Patient Consent

The New York Medical College IRB reviewed this study and deemed it exempt.

### Data Availability

Data not provided in the article because of space limitations may be shared (anonymized) at the request of any qualified investigator for purposes of replicating procedures and results.

## Results

### Preimplementation Needs Assessment Survey

A total of 12 responses were collected from among 33 eligible residents and attendings in the department (a combined 36% response rate). These responses were from 5 residents and 7 attendings, averaging 11.33 years in practice including residency training (range 1–34 years) (Table 1). In response to the primary research questions, 75% (9/12) of respondents strongly agreed or agreed that over the month before survey administration, they had at least 1 case where multispecialty input would have been beneficial for patient care. In total, 33% (4/12) of respondents strongly agreed or agreed that they had a good mechanism to discuss challenging cases within their own subspecialty, and 33% (4/12) felt the same regarding discussion with other subspecialties in neurology. In response to secondary, exploratory research questions, 17% (2/12) of respondents felt they had a good grasp of resources available at the institution (e.g., specialty laboratory tests, advanced imaging studies) outside their own subspecialty, and 33% (4/12) of respondents strongly agreed or agreed that they felt they knew who the right person at the institution was to answer questions about specific cases when they arose. The full data are presented in Table 1.

**Table 1:** Preimplementation Survey and 18-Month Follow-Up Postimplementation Survey Results

Questions	Presurvey	Postsurvey	p-value^b^
	N = 12^a^	N = 20^a^	
I am a, (%)			0.7
Attending	7 (58%)	13 (65%)	
Resident	5 (42%)	7 (35%)	
I have been in practice for ____ y (including residency)	5 (3, 21)	10 (3, 23)	0.7
Please state your level of agreement or disagreement with the following statements: Care in our department is siloed (%)			0.5
Agree	5 (42%)	6 (30%)	
Neither	4 (33%)	4 (20%)	
Disagree	3 (25%)	10 (50%)	
Please state your level of agreement or disagreement with the following statements: In the last mo, I have had at least 1 case where multispecialty input would have been beneficial to patient care (%)			0.3
Agree	9 (75%)	18 (90%)	
Neither	3 (25%)	2 (10%)	
Disagree	0 (0%)	0 (0%)	
Please state your level of agreement or disagreement with the following statements: I feel that I currently have a good mechanism to discuss challenging cases within my neurology subspecialty (%)			0.002
Agree	4 (33%)	18 (90%)	
Neither	6 (50%)	2 (10%)	
Disagree	2 (17%)	0 (0%)	
Please state your level of agreement or disagreement with the following statements: I feel that I currently have a good mechanism to discuss challenging cases with other subspecialists in neurology (%)			0.036
Agree	4 (33%)	16 (80%)	
Neither	3 (25%)	1 (5.0%)	
Disagree	5 (42%)	3 (15%)	
Please state your level of agreement or disagreement with the following statements: I know what resources we have across the institution, including what specialty laboratory tests, imaging studies, and other diagnostic studies we are able to order or perform here at WMC (%)			0.009
Agree	2 (17%)	14 (70%)	
Neither	5 (42%)	4 (20%)	
Disagree	5 (42%)	2 (10%)	
Please state your level of agreement or disagreement with the following statements: When I have a diagnostic question, I know who at our institution is the right person to answer it			0.062
Agree	4 (33%)	15 (75%)	
Neither	6 (50%)	4 (20%)	
Disagree	2 (17%)	1 (5.0%)	
How often do you refer patients to colleagues within the neurology department? (%)			0.14
Often	4 (33%)	12 (60%)	
Rarely	2 (17%)	0 (0%)	
Sometimes	6 (50%)	8 (40%)	
In the last 12 mo, I have been an author on ___ unique case reports with another trainee or faculty member that have been either submitted or accepted for presentation or publication (%)			0.6
0	4 (33%)	6 (30%)	
1	4 (33%)	8 (40%)	
2	4 (33%)	2 (10%)	
3	0 (0%)	2 (10%)	
4	0 (0%)	1 (5.0%)	
7	0 (0%)	1 (5.0%)	

Abbreviation: WMC = Westchester Medical Center.

a n (%); median (Q1, Q3).

b Fisher exact test; Wilcoxon rank-sum test.

### Postimplementation Survey

For the postimplementation survey, a total of 22 residents and attendings responded among 47 eligible residents and attendings in the department (a 46% response rate). The denominator grew due to expansion of the department. Two respondents were excluded due to lack of consent to participate in the survey and never participating in the conference, respectively. Of the remaining 20 respondents, 13 were attendings and 7 were residents, ranging from 1 to 61 years in practice. The frequency of attendance at the CCC among respondents ranged from those who attended rarely (1–3 times, 35%) to frequently (7–10 times, 20%) across a broad mix of platforms: in-person (45%), virtual (15%), or mixed (40%) attendance. More respondents (45%) had presented in the preassigned portion of the conference; 15% presented during the open forum.

The postimplementation data are presented in Table 1. After 18 months of CCC, respondents reported significant improvements in perceptions of available mechanisms for discussing challenging cases. The proportion of respondents who agreed or strongly agreed that they had a good mechanism to discuss challenging cases with other neurology subspecialties increased from 33% (4/12) at baseline to 80% (16/20) at follow-up, an absolute increase of 47 percentage points (*p* = 0.036). The proportion of respondents who agreed or strongly agreed that they had a good mechanism to discuss challenging cases within their own neurology subspecialties also increased from 33% (4/12) to 90% (18/20), an absolute increase of 57 percentage points (*p* = 0.002). There was no statistically significant difference in recognition in clinical cases where multispecialty input would be beneficial to patient care in the prior month (*p* = 0.3). In response to a secondary, exploratory research question, the percentage of respondents who agreed or strongly agreed to having an understanding and knowledge of the resources available at the institution (specialty laboratory tests, imaging studies, and other diagnostic studies) increased from 17% (2/12) to 70% (14/20), an absolute increase of 53 percentage points (*p* = 0.009). Secondary, exploratory questions that did not meet statistical significance included knowledge of who at the institution could help with a diagnostic question (33% (4/12) to 75% (15/20) respondents; *p* = 0.062) and tendency for referral to colleagues within the neurology department (33% (4/12) to 60% (12/20); *p* = 0.14).

We asked respondents to rate the factors which encouraged their attendance at CCC. Top reasons were (1) the conference was intellectually stimulating (75% of respondents), (2) it was an opportunity to consult fellow neurologists in different subspecialties on challenging cases (65% of respondents), (3) for learning opportunities and CME (60%), and (4) for networking opportunities with colleagues on different campuses (35% of respondents) (Table 2).

**Table 2:** Reasons for Respondents' Participation in the CCC

Question: Which factors encourage you to take the time to attend complex case conferences? (select all that apply)	N	% Of respondents
Intellectually stimulating	15	75%
To consult fellow neurologists in different subspecialties on challenging cases	13	65%
Learning opportunities (continuing medical education [CME])	12	60%
Networking opportunities with colleagues on different campuses	7	35%
To receive CME credit	5	25%
Joined for grand rounds and it happened to be a Complex Case Conference discussion	5	25%
Opportunity to practice presenting a case	3	15%
Required to attend	3	15%
Opportunity to write up a complex case as a case report	2	10%

Abbreviation: CCC = Complex Case Conference.

### CCC Implementation and Iterative PDSA Cycles

With iterative cycles month to month, changes were made in the CCC structure to better address the needs of the department and attendees at the conference. Initially, we envisioned a conference that would allow a mentee and mentor to present together. However, based on informal, early feedback from attendees at the CCC, attendings preferred to present their own cases. As a result, a rapid PDSA cycle was performed after 4 conferences with the mentor/mentee design, and the overall conference design was changed such that attendings would present their own cases and resident attendees would serve as observers and discussants to ask questions and offer thoughts and ideas.

In addition, a moderator was later introduced to support the flow of discussion and reinforce the time constraints within the two-phase format of CCC. The moderator also summarized key takeaway points at the end of each case discussed.

### Open Responses Identified the Benefits of Input From Colleagues

In an open response question, those who prepared a case to present ahead of time were asked, “How did input from colleagues help with determining next steps toward diagnosis of the patient?” Twelve responses were submitted. Themes in the responses suggested the CCC helped with outlining a differential, pursuing additional testing (e.g., imaging or biopsy), determining next steps in or changing management, or helping providers clarify their own thoughts or gain confidence in the management plan after hearing different perspectives and engaging in the group discussion.

## Discussion

Our study met its 2 overarching aims: first, our needs assessment demonstrated that there were limited formalized mechanisms for cross-specialty case discussion in our department, yet most respondents had cases that would benefit from such discussion; and second, that this need could be addressed by the existence of a structured, hybrid, monthly, department-wide case conference—the CCC. Our data demonstrate that CCC increased the perception that there was a good mechanism to discuss challenging cases with other neurology subspecialties and even within one's own subspecialty. As a secondary aim, the existence of CCC increased knowledge of what resources exist across the institution to be used in patient care. In this way, CCC provides one example of an intervention at the departmental level that can create space for collaborative discussion about patients whose needs cross-subspecialty boundaries and increase attendee knowledge in a tangible, practical manner.

A major goal of the CCC was to improve cross-specialty collaboration. In this way, the CCC was successful; forum participants showed a significant improvement in the perception of having a good mechanism to discuss cases across neurologic subspecialties. This suggests that the informal “curbside” to colleagues in other divisions may have been insufficient for clinician support on cases that crossed subspecialty boundaries. The need for improved communication was also not limited to trainees or junior attendings; the initial needs assessment included participants from a range of experience levels, yet demonstrated all participants across levels of expertise desired improved collaboration.

Through this forum, participants had the opportunity to meet and network with subspecialists at their institution, learning who they could go to for help with future questions. Participants also demonstrated a greater knowledge about institutional resources (*p* = 0.009). This suggests that one approach to addressing aspects of the general neurology gap may include forums that capitalize on a broad range of specialized knowledge and apply it to the patient in a team-based approach to care with an emphasis on diagnostic reasoning.

In a clinical landscape where comprehensive general neurology expertise is becoming increasingly limited, the CCC improved the perception of collaboration among attendings on tricky clinical cases. This is important, not just for clinical care but for physician growth and to help combat physician burnout.^16,17^ The difficulty of managing uncertainty within medicine is well-documented^16,18^; although most frequently studied in trainees,^17^ it remains a constant presence throughout a neurologist's career.^19^ Indeed, having a space to discuss challenging and clinically uncertain cases was widely regarded as necessary and valuable, from new neurologists one year into training to veteran neurologists several decades into their career. As an attending-driven forum, the CCC additionally serves as a teaching tool to collectively model for trainees how to behave in the face of clinical uncertainty^17^ and foster a judgement-free culture of growth at all stages of one's career. CCC also serves to emphasize the defining features of what it means to be a neurologist—collaborative diagnostic reasoning rooted in detailed history-taking, neuroanatomy, and pathophysiology.^20^ Although we did not formally evaluate these outcomes at this stage of initial implementation, these represent important future research avenues.

Throughout the 18 months of CCC, many changes were made to the structure of the program based on participant feedback. Initially, the forum was designed using an attending and resident mentor model where the assigned cases were presented by a trainee/attending combination; however, it was quickly realized that this turned the session more into a didactic lesson rather than a collaborative discussion. In addition, attendings preferred to present their own cases, focusing on the clinical questions giving them pause. To preserve the mission of the CCC, the forum subsequently was changed to have attendings and residents present their own cases. In addition, a moderator was added to the sessions to keep time and provide structure to the discussion. At the end of a case discussion, the moderator summarized the discussion and the main takeaways the physician presenting the case can bring back to the patient's care. This added structure provided a discrete, practical value to the physician and the patient.

There are several limitations to our study to consider. Given there is a high nonresponse rate, there is a risk of a nonrepresentative sample and nonresponse bias, and therefore, the results should be interpreted with these cautions. The needs assessment survey also did not use a prespecified cutoff to determine whether a true “need” existed. Instead, the authors considered a >50% majority to indicate sufficient need; the results should be interpreted in this context. In addition, although this work was intended to improve discussion among each subspecialty of neurology, residents were included as general neurologists, which may bias the findings in these data toward the null hypothesis. Further analyses will focus exclusively on subspecialty attending perspectives across different divisions and sites of care within the department.

This description of a new intervention focused on outcomes for clinical collaboration and multispecialty discussion. We did not report patient outcomes as a result of this forum. Finally, we share results from a single academic institution. Our results may not generalize to other academic centers which do not have similar geographic distribution or subspecialty divisions.

Future directions should also consider how forums such as the CCC improve clinical collaboration and patient care across geographically disparate networks, a growing need as healthcare systems expand and colleagues share physical office space less often. Preliminary data from our study suggested that the opportunity to network across different campuses was a driving factor in forum participation. We are also interested in evaluating appropriateness of referrals and diagnostic workup for patients because we hypothesize that this forum can streamline care for complex patients and lead to more efficient diagnoses (e.g., reducing redundant visits and inappropriate testing).

As each subspecialty within neurology drives forward in innovation, the field's complexity grows. It is becoming increasingly difficult for any single neurologist, generalist, or subspecialist to stay abreast of all the different diagnostic paradigms, studies, new diagnoses, and treatment options emerging across the field. This presents a day-to-day challenge for the practicing neurologist: as general neurology expertise becomes harder to come by, patients are often left trying to self-identify the right specialist to see and may need broader expertise than a single subspecialist can provide. The CCC proved to be a valuable method for amassing knowledge to benefit in these challenging cases.

In conclusion, we present data after 18 months of the CCC, a new effort to improve multisubspecialty clinical collaboration within an academic neurology department. These data demonstrate that creating a space for attendings to discuss clinical cases that benefit from cross-subspecialty input supports care collaboration, institutional knowledge sharing, and clinician growth.

## Glossary

ALS: amyotrophic lateral sclerosis
CCC: Complex Case Conference
IRB: Investigational Review Board
PFO: patent foramen ovale
WMC: Westchester Medical Center
